# Assessing the potential utility of commercial ‘big data’ for health research: Enhancing small-area deprivation measures with Experian™ Mosaic groups

**DOI:** 10.1016/j.healthplace.2019.05.005

**Published:** 2019-05

**Authors:** Welcome M. Wami, Ruth Dundas, Oarabile R. Molaodi, Mette Tranter, Alastair H. Leyland, Srinivasa Vittal Katikireddi

**Affiliations:** a MRC/CSO Social and Public Health Sciences Unit, 200 Renfield Street, University of Glasgow, Glasgow, G2 3AX, UK; b Directorate of Public Health and Health Policy, Lothian National Health Service (NHS) Board, Edinburgh, UK

**Keywords:** Commercial big data, Deprivation, Experian mosaic, Public health, Socioeconomic measures

## Abstract

In contrast to area-based deprivation measures, commercial datasets remain infrequently used in health research and policy. Experian collates numerous commercial and administrative data sources to produce Mosaic groups which stratify households into 15 groups for marketing purposes. We assessed the potential utility of Mosaic groups for health research purposes by investigating their relationships with Indices of Multiple Deprivation (IMD) for the British population. Mosaic groups showed significant associations with IMD quintiles. Correspondence Analysis revealed variations in patterns of association, with Mosaic groups either showing increasing, decreasing, or some mixed trends with deprivation quintiles. These results suggest that Experian's Mosaics additionally measure other aspects of socioeconomic circumstances to those captured by deprivation measures. These commercial data may provide new insights into the social determinants of health at a small area level.

## Highlights

• Mosaic groups showed a significant association with IMD quintiles.
• Trend patterns varied between different Mosaic groups across IMD quintiles.
• Mosaic groups have potential to enhance routinely used socioeconomic measures in research.

## 1 Introduction

Routinely available socioeconomic measures are needed for health services planning and research (Krieger et al., 2002; Galobardes et al., 2006). Area-based deprivation measures are widely used (e.g. the Index of Multiple Deprivation (IMD), Carstairs scores) since they are consistently related to both health and its determinants, are available for the whole population and are easily linked to many health datasets. However, they suffer significant limitations including being infrequently updated and area-based rather than individual measures (Fischbacher, 2014). IMD relies on benefits data which may no longer be available after welfare reform in the UK, while the potential abolition of the decennial census threatens the future of Carstairs scores (Office for National Statistics, 2015). Furthermore, neither measure is designed to assist intervention targeting but are often used in this way (Mcloone, 2001; Katikireddi and Valles, 2015).

There is considerable research, policy and practice interest in alternative socioeconomic measures (Fischbacher, 2014; Doos et al., 2014). ‘Big data’ from the commercial sector could act as an alternative to deprivation measures and provide new insights for research and practice. Presently, there is huge demand for health intelligence to inform local decision-making by public health practitioners (Wang and Desalvo, 2018). In addition, the global need for population-specific socio-economic indicators for predicting health outcomes and studying inequalities at smaller geographies, for example, using measures such as Geodemographics has been highlighted in several recent international studies (Lopez-De Fede et al., 2016; Berkowitz et al., 2015; Halonen et al., 2013; Havard et al., 2008; Cabrera-Barona et al., 2015). This alternative source of information might not only act as an alternative to deprivation measures, but also facilitate the investigation of novel targets for intervention or the development of new measures that facilitate monitoring of local areas (Doos et al., 2014; Farr et al., 2008). The latter issue is particularly pertinent at present, given the need for local data that are amenable to monitoring at regular intervals to guide the actions of public health activity located in local authorities in England and Health and Social Care Partnerships in Scotland.

Briefly, geodemographic classification groups areas into categories based on shared socioeconomic characteristics (Webber, 2004). The foundation of geodemographic classification techniques is mainly based on the idea of ‘linking people to places’ considering factors such as their physical, behavioural, social and economic properties (Harris et al., 2005; Parker et al., 2007; Singleton and Spielman, 2013). There is a wide variety of commercial geodemographic classifications developed around the world and adopted mainly as a tool for strategic marketing in the private sector (Harris et al., 2005). Two other closely comparable examples include ARCON (A Classification of Residential Neighbourhoods) in the UK and PRIZM (Potential Rating Index for ZIP Markets) in the USA (Flowerdew and Goldstein, 1989; Batey et al., 2008). A review by Singleton and Spielman (2013) gives a more detailed overview and historical comparison of these geodemographics and their use. The interest in the development of Geodemographics has also extended to the public sector. For example, the Output Area Classification (OAC) has been developed in the UK (first in 2001 and subsequently in 2011) as an open source public geodemographic classification built entirely from decennial census data (Gale et al., 2016). Several case studies have shown that geodemographics have some practical advantages by incorporating a wide range of data sources, hence can be useful tools to inform policy (Harris et al., 2005; Parker et al., 2007).

Experian is one such commercial company that collates diverse information to produce socio-demographic and lifestyle variables, primarily for marketing purposes (Experian Ltd, 2015). Unlike traditional deprivation measures, these variables are designed to reflect affluence and consumption patterns. The use of Experian's geodemographic segmentation (Mosaics) has recently been slowly gaining popularity in health research in the United Kingdom (UK), often used to identify health risk factors at local community level (Douglas and Szatkowski, 2013; Sharma et al., 2010; Iyen-Omofoman et al., 2011; Doos et al., 2014). But the use of Mosaic geodemographic classification has not been limited to the UK only. For example, similar studies using country-specific Mosaic classification have been conducted in Japan (Kimura et al., 2011), Italy (Willis et al., 2014), Sweden (Sundberg et al., 2015), and the USA (Hohl et al., 2006; Lopez-De Fede et al., 2016). However, there remains a need to gain a deeper insight into the characteristics of these commercial sector data and how they compare to the more commonly used socioeconomic measures. The aim of this study was to determine the potential utility and feasibility of combining commercial data and routine socioeconomic measures in research for the purposes of understanding population health. Thus, to fill this gap in knowledge, we assessed the overlap and patterns of association between Experian's Mosaic classification (groups) of the British population and the widely used measures of deprivation, that is, IMD and its devolved equivalents for Scottish (SIMD) and Welsh (WIMD) populations.

## 2 Materials and methods

### 2.1 Ethical issues

This research study was conducted on secondary data, with approval already obtained from all the data sources and raised no new ethical concerns. Further advice was sought from [BLANKED FOR PEER REVIEW] the relevant Research Ethics Committee, who confirmed that the study did not require ethical approval. Due to reasons of commercial confidentiality, the investigators did not have access to any individual-level information and detailed methods used to create Experian's Mosaic groups.

### 2.2 Data sources

This study utilised data from two different sources: (i) the public sector held official measures of area deprivation based on the 2011 census data defined for Lower-layer Super Output Areas (LSOAs) in England (Department for Communities and Local Government, 2010) and Wales (Welsh Government, 2011), and Data Zones in Scotland (Scottish Government, 2012); (ii) the commercial sector Mosaic data sourced from ^©^ 2016 Experian Limited, available at full postcode level.

### 2.3 Measures of area deprivation

The Index of Multiple Deprivation (IMD) is currently the official measure of relative deprivation for England, with equivalents available in the other constituent countries of the UK (Department for Communities and Local Government, 2015). It is widely used by central and local government, the National Health Service (NHS), and third sector to distribute funding, target resources or prioritise delivery of interventions or services to areas. The history of indices of deprivation in the UK dates back as far as mid-1960s, created with the aim to improve the effectiveness of target programmes (Noble et al., 2006). Previous indices of area deprivation comprised of a small number of indicators (Noble et al., 2006) and relied heavily on Census data, which meant they quickly became outdated (Department for Communities and Local Government, 2015). In 2000 in England and Wales, and 2003 in Scotland, the Indices of Deprivation were refined to include multiple deprivation measures. They consist of a broad range of updatable domains that measure different aspects of deprivation, drawn extensively on data from administrative records rather than solely relying on Census measures only (Brown et al., 2014; Fairburn et al., 2016; Norman, 2016). Since then, subsequent updates in the construction of these indices in the UK constituent countries have been implemented every 3–4 years. In addition, there have been changes in the number of indicators used and the level of geography for reporting IMDs, for example, from Wards to a much finer spatial scale such as LSOAs in England and Wales (Department for Communities and Local Government, 2015).

The English IMD comprises seven domains relating to: employment, income, health and disability, education skills and training, barriers to housing and services, crime and disorder, living environment (Department for Communities and Local Government, 2010). For this study, we considered the English IMD 2010 version, based on data from 2008 to 2010. LSOAs are the smallest spatial units for which the English IMD is defined, consisting of 32,844 LSOAs, with an average population of 1500 people (Department for Communities and Local Government, 2015). The Welsh IMD uses eight domains: income, health, employment, education, access to services, community safety, physical environment, and housing. For WIMD 2011, based on data between 2008 and 2010, a total of 1896 LSOAs were ranked by relative deprivation, with an average population size of 1600 people (Welsh Government, 2011). The Scottish IMD comprises seven domains of deprivation: income, employment, education, housing, health, crime, and access to amenities and services (Scottish Government, 2012). We used the SIMD 2012 version, based on data covering periods 2010–2012. For SIMD, Data Zones (n = 6976) are the available key geography for small area statistics in Scotland, with an average population of 800 people per Data Zone (Scottish Government, 2015).

### 2.4 Experian Mosaic data

Mosaic is Experian's consumer geodemographic classification of the population into a number of different ‘like-minded’ groups based on individual characteristics directly linked to every household, postcode, retail catchment and local area across the UK (Farr and Webber, 2001; Experian Ltd, 2015). Experian describes Mosaic segmentation as “a process that combines more than 850 million source records with 450 + variables to fully understand consumer preferences” (Experian Ltd, 2015). The wide range of data sources used by Experian to create Mosaic segmentation includes: Census data, Office for National Statistics (ONS) local area statistics, Electoral Rolls, House Price and Council Tax information, Consumer Credit Activity, and self-reported demographics and consumer behaviour from marketing surveys. Thus, Mosaic classifications go beyond the standard demographic characteristics, providing further insights such as education, health and lifestyle choices, purchasing behaviours, family composition, occupational details, and location of individuals and households in the geographic area. The available Experian Mosaic data were created in 2014 using data from the period 2010–2014 and classify all UK consumers into 66 distinct lifestyle types and 15 groups which aim to comprehensively describe their socioeconomic and sociocultural behaviour (Experian Ltd, 2015). For the purposes of this study, we considered only the Mosaic groups within the Experian dataset for analyses, available at full residential postcode for Great Britain (GB). Each unique postcode was allocated to one and only one Mosaic group. A description of the 15 Mosaic groups is given in Table 1. In addition, a separate Mosaic classification for Scotland (hereafter Mosaic Scotland) was considered, also built in 2014, recognising the different socioeconomic, demographic, lifestyle, and behaviour in Scotland compared to the rest of the UK (see Supplementary Information, Table S1). Residential postcodes within the Experian Mosaic dataset were linked to the 2011 LSOAs or Data Zones and then to the measures of area deprivation (IMD, SIMD and WIMD).

**Table 1:** Description and key features of Experian's Mosaic Groups.

Mosaic Group	Description	Key features
A: City Prosperity	High status city dwellers living in central locations and pursuing careers with high rewards	Highly educated; High value properties; Central city areas; High status jobs; Charity membership; High Internet use
B: Prestige Positions	Established families in large detached homes living upmarket lifestyles	Likely to be 56–75 years old-Well-educated; High value detached homes; Married couples; Charity membership; Strongly motivated by religious beliefs; High assets and investments; Online shopping and banking
C: Country Living	Well-off owners in rural locations enjoying the benefits of country life	Charity membership; Well-off homeowners; Attractive detached homes; Higher self-employment; Support environmental causes; High use of Internet
D: Rural Reality	Householders living in inexpensive homes in village communities	Aged most likely between 46 and 55 years-Support the community; Donate to charity shop; Agricultural employment; Most are homeowners; Affordable value homes; Slow Internet speeds
E: Senior Security	Elderly people with assets who are enjoying a comfortable retirement	Aged average 75+-Elderly singles and couples; Homeowners; Donate on a regular basis; Additional pensions above state; Don't like new technology; Strongly motivated by religious beliefs
F: Suburban Stability	Mature suburban owners living settled lives in mid-range housing	Aged 45 to 65-Older families; Some adult children at home; Suburban mid-range homes; Likely to donate soon; Donate low amounts; Research on Internet
G: Domestic Success	Thriving families who are busy bringing up children and following careers	Aged late 30s–40s-Families with children; Upmarket suburban homes; Support a friend through sponsorship; Support Health and medicine; High Internet use; Own new technology
H: Aspiring Homemakers	Younger households settling down in housing priced within their means	Age 20s & 30s-Younger households; Full-time employment; Support a friend through sponsorship; Affordable housing costs; Starter salaries; Willingness to donate
I: Family Basics	Families with limited resources who have to budget to make ends meet	Aged 25 to 40-Families with children; Limited charitable activity; Cannot afford to give to charity; Some rent from social landlords; Squeezed budgets
J: Transient Renters	Single people privately renting low cost homes for the short term	Age 20s & 30s; Private renters,; Low length of residence; Low cost housing; Singles and sharers; Prompted by colleague at work/school; Support Animal Welfare
K: Municipal Challenge	Urban renters of social housing facing an array of challenges	Social renters; Working age; Donate small amounts or nothing; Feel the state does not help those in need; Few employment options; Low income; Mobile phones
L: Vintage Value	Elderly people reliant on support to meet financial or practical needs	Aged 74 average-Elderly; Living alone; Low income; Unlikely to donate; Support traditional British charities; Low technology use
M: Modest Traditions	Mature homeowners of value homes enjoying stable lifestyles	Aged between 46 & 65-Mature; Homeowners; Affordable housing; Unlikely to donate; Interested in animal welfare; Modest income
N: Urban Cohesion	Residents of settled urban communities with a strong sense of identity	Aged 18–35; Private renting; Singles and sharers; Support Human rights; Support a friend through sponsorship; High use of smartphones
O: Rental Hubs	Educated young people privately renting in urban neighbourhoods	Aged 18–35; Private renting; Singles and sharers; Support Human rights; Support a friend through sponsorship; High use of smartphones

### 2.5 Statistical analysis

The Cochrane-Armitage test for trends (Armitage, 1955) was used to assess relations between each of the Mosaic groups (nominal variables) and deprivation quintiles (ordinal variables). To summarise and interpret the patterns of association among the different Mosaic groups (15-levels), as well as between the measures of deprivation quintiles (5-levels), we used Correspondence Analysis (Greenacre, 1984). Briefly, Correspondence Analysis (*CA*) is a statistical technique for visualising graphically the rows and columns of a contingency table as points in a reduced dimensional space (Biplot), such that “the positions of the row and column points are consistent with their associations in the table” (Kassambara, 2017). For each variable, if a category profile is different from the average group profile (centroid), then the point will lie far from the origin whereas profiles that are close to the average are represented by points close to the centroid. If all categories have equal profiles, then all points will lie in the centroid (i.e. indicating there is no difference between the categories of a row or column variable being profiled). Thus, *CA* facilitates understanding of patterns among categorical variables of a large dataset (Greenacre, 2007). In addition, a strong association between a row and column level will be indicated by a small angle connecting them to the origin. In all the analyses in this study, the CA was based on symmetrically normalised data. To ensure correct interpretation of the results, we first determined the appropriate number of dimensions to describe the associations by examining the percentage of total variation explained in the analysis. We used the R package “ca” to compute and visualise correspondence patterns by means of Biplot maps (Nenadi and Greenacre, 2007).

## 3 Results

### 3.1 Postcode Mosaic coverage by Experian

**Fig. 1:**
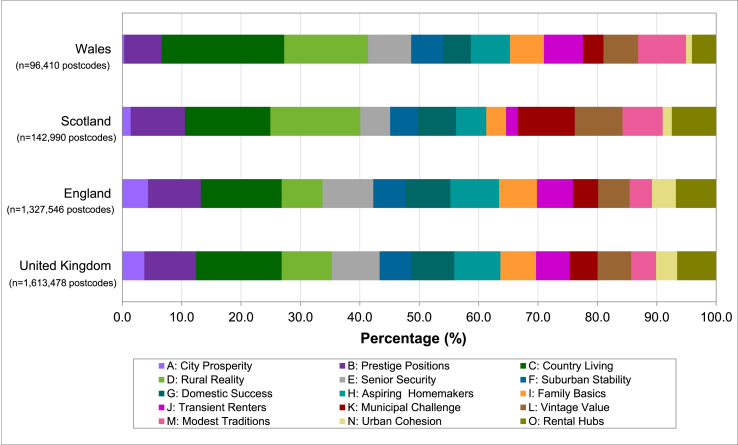
Percentage distribution of Mosaic groups.

Of the 1.7 million postcodes in GB, 92% were classified into different Mosaic groups by Experian. The unclassified postcodes were non-residential, typically for business or large (mail) users and these were not included in the final analyses. Fig. 1 shows the percentage distribution of residential postcodes by Mosaic group profiles successfully included within the Experian's geographic segmentation dataset. Of the 15 Mosaic groups, ‘Country Living’ was identified as the most common Mosaic in all three constituent countries in GB, accounting for more than 14% of postcodes. ‘Urban Cohesion’ was among the least represented Mosaic groups and comprised less than 2% of postcodes in Scotland and Wales (Fig. 1). Furthermore, variations in the distribution of some Mosaic groups across the three countries were observed. Notably, ‘Municipal Challenge’ and ‘Vintage Value’ were much more common in Scotland compared to the rest of GB. On the other hand, ‘City Prosperity’ was most popular among postcodes in England only. ‘Modest Traditions’ was relatively more common in Wales as compared to England and Scotland. Similar distribution patterns were observed for Mosaic Scotland groups. For example, ‘Country Living’ and ‘Rural Reality’ were identified as the most common, with ‘City Prosperity’ being the least frequent group in the Scottish population (Supplementary Information, Table S2). Furthermore, we explored the distribution of Mosaic in terms of LSOAs or Data Zones by means of a summary table showing the number and percentage of LSOAs/Data Zones with different Mosaic groups in them (Supplementary Information, Table S3). Scotland showed more homogeneity than England and Wales (that is, more Data Zones with <4 Mosaic groups compared to LSOAs).

### 3.2 Distribution patterns of Mosaic groups across deprivation quintiles

**Fig. 2:**
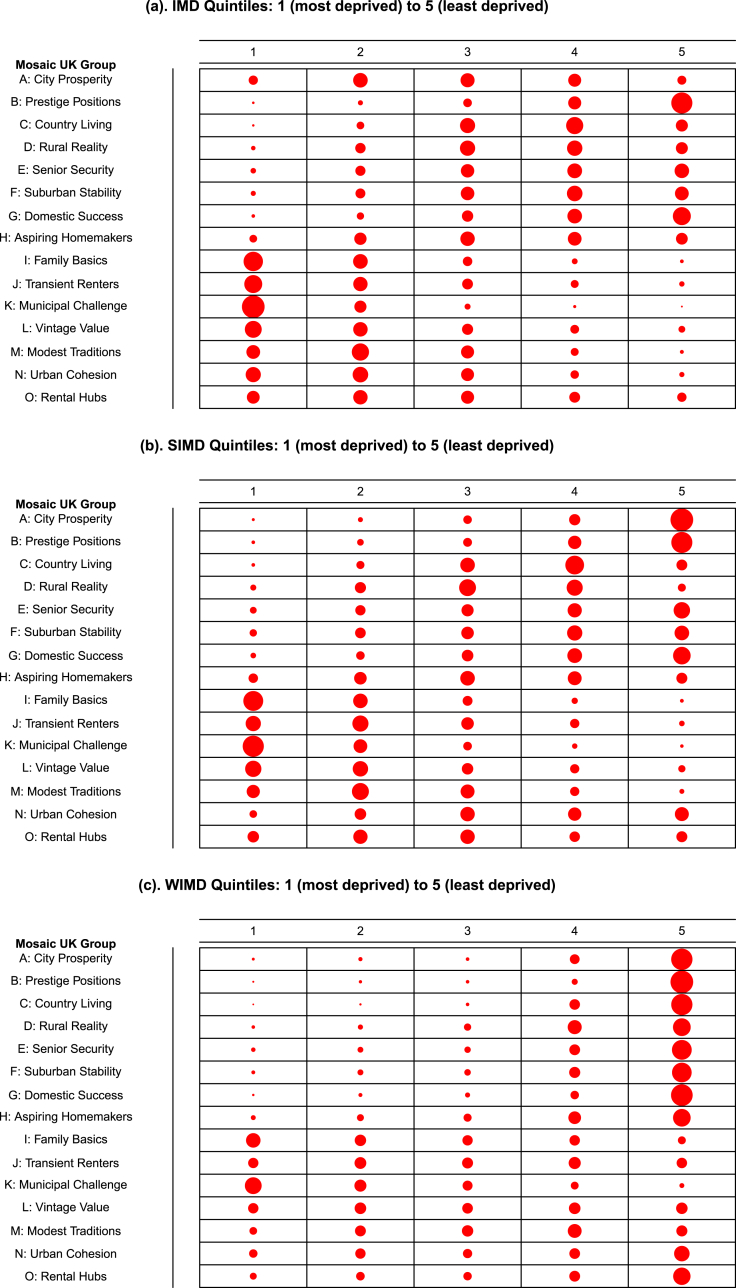
**Bubble charts of Experian Mosaic group profiles by Deprivation quintiles.** Dot size is proportion proportional to the percentage cell frequency. (a) IMD percentage range: 0.1–75.9%, (b) SIMD percentage range: 0.5–72.8%, (c) WIMD percentage range: 0.1–93.1%.

Fig. 2 shows bubble charts to graphically explore the relationship between Mosaic groups and deprivation measures, and the bubble size is proportional to the number of respective Mosaic groups captured within each deprivation quintile (i.e. cell frequency in the cross-tabulation). Assessing patterns of bubble sizes from the plots revealed interesting varying trends. Firstly, the proportion of some Mosaic groups either showed a consistently increasing trend (for example, ‘Family Basics’, ‘Transient Renters’, ‘Municipal Challenge’ and ‘Vintage Value’) or decreasing trend (for example, ‘Prestige Positions’ and ‘Domestic Success’) with deprivation quintiles, suggesting a strong relationship with levels of area deprivation. Secondly, there were Mosaic groups (for example, ‘Modest Traditions’ and ‘Rental Hubs’) that showed mixed patterns indicating some variations in their relationship with deprivation. When focussing on Scotland only (that is, using Scotland Mosaic groups), similar patterns were found (Supplementary Information, Fig. S1). The Cochrane-Armitage test for trend revealed significant differences (all *P* < 0.001) in the distribution of Mosaic groups across the levels of deprivation measures, except for the ‘Aspiring Homemakers’ Mosaic for the Scottish population ($\chi^{2}$ trend = −1.60; *P* = 0.107).

### 3.3 Relative associations between Mosaic groups and measures of deprivation

**Fig. 3:**
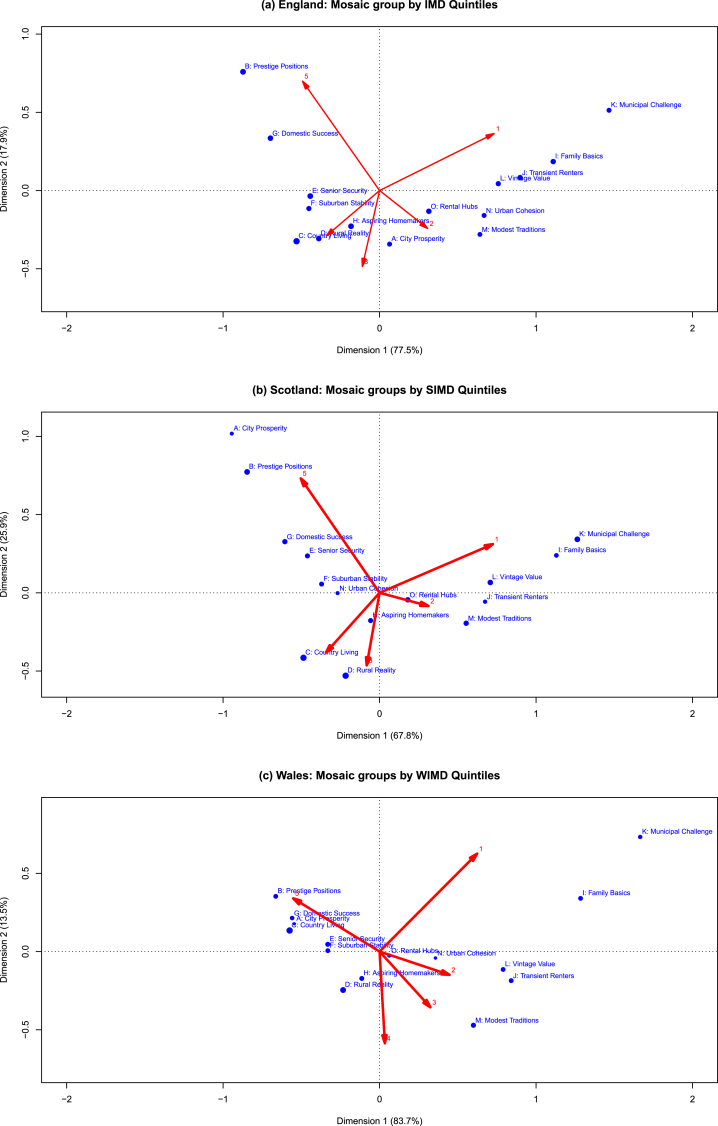
**Correspondence Analysis Biplot maps to assess relative patterns of associations between Mosaic groups and measures of Deprivation quintiles.** (a) IMD, (b) SIMD, (c) WIMD. The red arrows indicate the deprivation quintiles.

Correspondence Analysis revealed that two dimensions were adequate for the interpretation of the relationships between Mosaic groups and measures of deprivation quintiles, explaining 95% of total variation for England, 94% for Scotland, and 97% for Wales (Fig. 3). The proximity of ‘Municipal Challenge’ and ‘Family Basics’ to the 1st deprivation quintile (shown by the small angle formed when connecting them back to the origin) in Fig. 3 indicated that these Mosaic groups were strongly associated with high levels of area deprivation. In addition, in Scotland, the ‘Family Basics’ Mosaic group trended positively with increasing levels of deprivation. Likewise, the proximity of ‘Prestige Positions’ (true for all the three constituent countries) and ‘Domestic Success’ (for England and Wales) with quintile 5 indicates suggested that these particular Mosaics were mostly associated with decreasing levels of area deprivation relative to other groups. However, not all Mosaic groups consistently trended with increasing/decreasing levels of area deprivation. For example, closely assessing the positions of ‘Urban Cohesion’ and ‘Aspiring Homemakers’ relative to deprivation quintiles in the Biplot correspondence map (Fig. 3), the suggested profiles of these Mosaic groups seemed to measure other additional aspects of socioeconomic circumstances to those directly captured by the measures of deprivation. Also of note, ‘Rental Hubs’ Mosaic was much closer to the origin (particularly for Scotland and Wales), suggesting a weak correspondence with any of the deprivation quintiles. When focussing on the Scottish population only, the analysis of Mosaic Scotland groups revealed consistent patterns with the ones described above (Supplementary Information, Fig. S2).

## 4 Discussion

### 4.1 Main findings

Our study showed the proportion of Mosaic groups varied across deprivation levels, with some Mosaics showing consistently increasing or decreasing patterns, while others showed mixed trends (for example an increase followed by a decrease) with IMD quintiles. Using the method of Correspondence Analysis, we were able to simultaneously describe the relationships between the different Mosaic groups, as well as the nature and strength of their association with measures of area deprivation. The different Mosaic groups spread over the resultant Biplot map rather than clustering together, indicating the extent of similarity/dissimilarity in terms of the socioeconomic aspects they capture. Importantly, some Mosaic groups showed strong patterns of association with deprivation quintiles. However, not all Mosaic groups seemed to be differential relative to area deprivation in this population. For example, the results suggested that ‘Rental Hubs’ Mosaic was less distinct in terms of the socioeconomic aspects it captured. A plausible explanation for this finding is that this particular Mosaic predominantly consists of student population who tend to reside in locations closer to educational institutes, hence it is less likely to reveal any consistent patterns in relation to levels of area deprivation.

### 4.2 Meaning of the study: possible implications for research or policymakers

Important implications for research and policy can be drawn from our findings. Emphasis on local provision is becoming important in public health practice in many countries, including England, as reflected by move of Public Health into local authorities (Department of Health, 2010; Smith and Hellowell, 2012). This increasing interest in place-based approaches in small areas requires more detailed area measures. Commercial data like Experian's Mosaics may address this need. The findings showing that some Mosaics captured different aspects to those measured by conventional measures of deprivation may facilitate precision in public health by targeting behaviour change interventions and improved preventive measures. Closely related to the above point, deprivation is a widely used socioeconomic position measure in health research. However, Mosaics may help in the investigation of other social characteristics – particularly since they include very detailed data in their development, such as loyalty card information and credit card transactions. Furthermore, they can help address residual confounding in research since many analyses (particularly for a growing number of studies that rely exclusively on administrative data) only include a single deprivation measure variable. Mosaics and associated data are often available on a more timely basis and also at a range of spatial scales – including at the individual and household levels. This may help with monitoring the determinants of health, for example trends in social patterning of smoking in the UK (Douglas and Szatkowski, 2013) and targeted prevention in the USA (Lopez-De Fede et al., 2016). Lastly, some key variables underpinning deprivation measures are under threat in the UK, for example, the abolition of free school meals, introduction of Universal Credit, potential abolition of census. Commercial data could help fill this gap. Furthermore, commercial data could facilitate international comparisons in the future.

### 4.3 Our findings in relation to other studies

This study broadly supports the work of other studies in this area, demonstrating that socioeconomic measures such as Experian's Mosaic can be effectively linked with existing databases for health research purposes (Doos et al., 2014). For example, Lopez-De Fed and colleagues in their study using linked data in the USA showed that an alternative small-area socioeconomic index performed better at predicting chronic disease burden compared to the commonly used measures such as the Townsend index of material deprivation (Lopez-De Fede et al., 2016). In addition, our findings are closely consistent with a recent study which also showed an association between Mosaics and relative deprivation in relation to smoking behaviours (Sharma et al., 2010). Taken together, our results showing varying patterns of correspondence suggest that the Experian's Mosaics offer something different, or even extra, in terms of aspects of socioeconomic circumstances to that already measured by IMD. These results corroborate the findings from a study by Petersen and colleagues, who also demonstrated the potential of Local Authority geodemographic classifications as valuable alternative tools for targeted public health neighbourhood interventions in England (Petersen et al., 2011). Furthermore, our study adds an extra dimension to what is already known. We explored the patterns of associations between each of the different Mosaics, as well as in relation to levels of IMD. This approach could help in identifying specific Mosaic groups that could closely be used to enhance area-level deprivation measures in targeted interventions or facilitate a more refined analytic approach for future work when using these socioeconomic measures as predictors of health outcomes such as mortality in the population.

### 4.4 Strengths and limitations of this study

One of the major strengths of Experian's Mosaic classification as used in this study is that the population level data are available at a finer level of geography (that is, full postcode) and updated more regularly than IMD. The Mosaics are therefore less likely to be influenced by area heterogeneity, allowing deeper insights into the characteristics of the population, their service needs and the health challenges they face at local community level (Petersen et al., 2011). In our study we demonstrated the potential of Mosaics in enhancing the existing measures of area deprivation using the method of Correspondence Analysis (CA). One strength of this analytic approach is that CA is conducted at the level of the response categories rather that at variable level, hence it preserves the categorical nature of the variables being analysed (Sourial et al., 2010). Furthermore, CA does not require underlying distributional assumptions, thus accommodating the different types of categorical variables being investigated in our study, that is, nominal variable (Mosaic groups) *vs.* ordinal variable (deprivation quintiles).

While acknowledging Mosaic measures affluence (Webber, 2004; Petersen et al., 2011) and IMDs measure deprivation (Deas et al., 2003; Noble et al., 2006; Batey and Brown, 2007), the aim was to determine any added benefit of using Mosaic over IMDs for policy, planning and research. However, there are issues inherent in the use of Mosaic data that must be taken into consideration when interpreting the results of our findings. Firstly, the theoretical difference emanating from the different concepts that IMDs and Experian Mosaic datasets aim to measure may result in inconsistencies in patterns of association. An example of this phenomenon was noted for the ‘Rental Hubs’ Mosaic as described earlier. Secondly, another important issue to consider is that the two measures explored in this study were created at different spatial aggregations, that is, the IMDs at LSOA/Datazone vs. Mosaic at full postcode. Consequently, this limits the extent to which one can fully and conclusively evaluate the extent of heterogeneity of Mosaic Groups within a LSOA/Data Zone using these data. Thirdly, there is a degree of overlap in the sources of data used as inputs IMDs and Mosaic, hence the possibility of this driving these two different measures to correspond cannot be ruled out. Fourthly, it should also be acknowledged that later versions of IMDs (2014/15/16) would have been an alternative possibility to use, however, although the Experian Mosaic were released in 2014, much of the input data was from 2010 onwards and hence we did not consider this approach in this present study. Lastly, access to Experian data is still very limited and not free for commercial reasons in contrast to IMD data which are readily available and are easily linked to many health datasets. This, combined with other factors such as lack of transparency in the methodology employed by Experian in deriving Mosaic can be a limitation to the widespread use of these data in health-related research and its uptake by researchers and policy makers. The pricing of these commercial data varies with the level of detail requested and fall within the core business of Experian. Enquiries about the estimated costs can be obtained from the Experian's Marketing Services.

## 5 Conclusions

Our investigation showed that the Experian's Mosaic profiles were not captured in only one IMD quintile, but the patterns of association across deprivation levels varied between different Mosaic groups. In conclusion, the finding of this study strengthens the idea that Mosaics can be used to enhance routinely used socioeconomic measures such as small-area deprivation indices in health-related research. These commercial sector data thus may provide new insights into the social determinants of health at small area level. Further research could usefully explore how these commercial data compare against the alternative open source public geodemographics such as the OAC.

## Declaration of interest

The study investigators do not have any direct financial conflicts of interest. However, we acknowledge there is the potential for such issues to arise in this study given the collaboration required with a private sector company. Consideration has been taken to address these issues in detail to minimise the risks of any adverse impacts on the quality of the research. First, the researchers' institution and ^©^ 2016 Experian Limited had a contract drawn prior to the work to be conducted. This was to assure the freedom of the researchers in terms of both study conduct and also crucially dissemination of results. The agreement between the researchers' institution and ^©^ 2016 Experian Limited stipulates that, for reasons of commercial confidentiality, the investigators will not have access to the detailed means of how the Mosaic classifications were created. As a consequence, the study team did not have access to any potentially highly sensitive individual-level information which the company holds. The intellectual property of the ^©^ 2016 Experian Limited data will remain with the company.

## Funding source

This work was supported by the Medical Research Council [MC_UU_12017/13; MC_UU_12017/15] and the Chief Scientist Office of the Scottish Government [SPHSU13; SPHSU15]. SVK was supported by a NRS Senior Clinical Fellowship (SCAF/15/02). The funding sources had no involvement in study design; in the collection, analysis and interpretation of data; in the writing of the report; and in the decision to submit the article for publication.
