# Supplementary material for: Assessing the potential utility of commercial ‘big data’ for health research: Enhancing small-area deprivation measures with Experian™ Mosaic groups
Source: Health Place. 2019 May;57:238–46. doi: 10.1016/j.healthplace.2019.05.005 (PMC6686722; doi:10.1016/j.healthplace.2019.05.005)
Supplement: Multimedia component 1 [file mmc1.docx]

**Supplementary Information**

**Table S1: Description and key features of the Experian’s Mosaic Scotland groups**

| **Mosaic Scotland Group** | **Description** | **Key features** |
| --- | --- | --- |
| A: City Prosperity | High status city dwellers living in central locations and pursuing careers with high rewards | High value properties; Central city areas; High status jobs; Charity membership; Strongly believe in charity; High Internet use |
| B: Prestige Positions | Established families in large detached homes living upmarket lifestyles | High value detached homes; Married couples; Charity membership; Strongly motivated by religious beliefs; High assets and investments; Online shopping and banking |
| C: Country Living | Well-off owners in rural locations enjoying the benefits of country life | Charity membership; Well-off homeowners; Attractive detached homes; Higher self-employment; Support environmental causes; High use of Internet |
| D: Rural Reality | Householders living in inexpensive homes in village communities | Support the community; Donate to charity shop; Agricultural employment; Most are homeowners; Affordable value homes; Slow Internet speeds |
| E: Senior Security | Elderly people with assets who are enjoying a comfortable retirement | Elderly singles and couples; Homeowners; Donate on a regular basis; Additional pensions above state; Don't like new technology; Strongly motivated by religious beliefs |
| F: Suburban Stability | Mature suburban owners living settled lives in mid-range housing | Older families; Some adult children at home; Suburban mid-range homes; Likely to donate soon  Donate low amounts; Research on Internet |
| G: Domestic Success | Thriving families who are busy bringing up children and following careers | Families with children; Upmarket suburban homes; Support a friend through sponsorship; Support Health and medicine; High Internet use; Own new technology |
| H: Aspiring Homemakers | Younger households settling down in housing priced within their means | Younger households; Full-time employment; Support a friend through sponsorship; Affordable housing costs; Starter salaries; Willingness to donate |
| I: Family Basics | Families with limited resources who have to budget to make ends meet | Families with children; Aged 25 to 40; Limited charitable activity; Cannot afford to give to charity; Some rent from social landlords; Squeezed budgets |
| J: Transient Renters | Single people privately renting low cost homes for the short term | Private renters; Low length of residence; Low cost housing; Singles and sharers; Prompted by colleague at work/school; Support Animal Welfare |
| K: Municipal Challenge | Urban renters of social housing facing an array of challenges | Social renters; Donate small amounts or nothing; Feel the state does not help those in need; Few employment options; Low income; Mobile phones |
| L: Vintage Value | Elderly people reliant on support to meet financial or practical needs | Elderly; Living alone; Low income; Unlikely to donate; Support traditional British charities; Low technology use |
| M: Modest Traditions | Mature homeowners of value homes enjoying stable lifestyles | Mature age; Homeowners; Affordable housing; Unlikely to donate; Interested in animal welfare  Modest income |
| N: Urban Cohesion | Residents of settled urban communities with a strong sense of identity | Aged 18-35; Private renting; Singles and sharers; Support Human rights; Support a friend through sponsorship; High use of smartphones |
| O: Rental Hubs | Educated young people privately renting in urban neighbourhoods | Aged 18-35; Private renting; Singles and sharers; Support Human rights; Support a friend through sponsorship; High use of smartphones |

***Source:*** © 2016 Experian Limited., Mosaic UK Generation 6 Data Profile. Available Online: [www.experian.co.uk/marketing-services](http://www.experian.co.uk/marketing-services). [Accessed 13 December 2017]

**Table S2: Frequency Distribution of Mosaic Scotland Groups.**

| **Mosaic Scotland Group** | **Number of postcodes** | **Percent (%)** |
| --- | --- | --- |
| A: City Prosperity | 3,957 | 2.77 |
| B: Prestige Positions | 11,060 | 7.73 |
| C: Country Living | 20,682 | 14.46 |
| D: Rural Reality | 21,663 | 15.15 |
| E: Senior Security | 7,348 | 5.14 |
| F: Suburban Stability | 8,574 | 6.00 |
| G: Domestic Success | 9,299 | 6.50 |
| H: Aspiring Homemakers | 7,527 | 5.26 |
| I: Family Basics | 4,803 | 3.36 |
| J: Transient Renters | 7,092 | 4.96 |
| K: Municipal Challenge | 8,370 | 5.85 |
| L: Vintage Value | 11,503 | 8.04 |
| M: Modest Traditions | 9,897 | 6.92 |
| N: Rental Hubs | 11,215 | 7.84 |
| **Total** | **142,990** | **100.0** |

**Table S3: Number and proportion of LSOAs or Data Zones with different Experian Mosaic groups in them.**

| **England** | | | **Scotland** | | **Wales** | |
| --- | --- | --- | --- | --- | --- | --- |
| **No. of different Mosaic Groups** | **No. of LSOAs** | **Percent** | **No. of Datazones** | **Percent** | **No. of LSOAs** | **Percent** |
| **1** | 709 | 2.2 | 352 | 5.1 | 12 | 0.7 |
| **2** | 2,010 | 6.1 | 998 | 14.3 | 202 | 10.9 |
| **3** | 3,332 | 10.1 | 1,149 | 16.5 | 118 | 6.4 |
| **4** | 4,447 | 13.5 | 1,281 | 18.4 | 168 | 9.1 |
| **5** | 5,200 | 15.8 | 1,148 | 16.5 | 265 | 14.4 |
| **6** | 5,077 | 15.5 | 842 | 12.1 | 273 | 14.8 |
| **7** | 4,680 | 14.3 | 577 | 8.3 | 267 | 14.5 |
| **8** | 3,586 | 10.9 | 338 | 4.9 | 225 | 12.2 |
| **9** | 2,139 | 6.5 | 187 | 2.7 | 157 | 8.5 |
| **10** | 1,112 | 3.4 | 68 | 1.0 | 97 | 5.3 |
| **11** | 411 | 1.3 | 24 | 0.3 | 41 | 2.2 |
| **12** | 123 | 0.4 | 7 | 0.1 | 20 | 1.1 |
| **13** | 18 | 0.1 | 3 | 0.04 | 2 | 0.1 |
| **Total** | **32,844** | **100.0** | **6,974** | **100** | **1,847** | **100.0** |


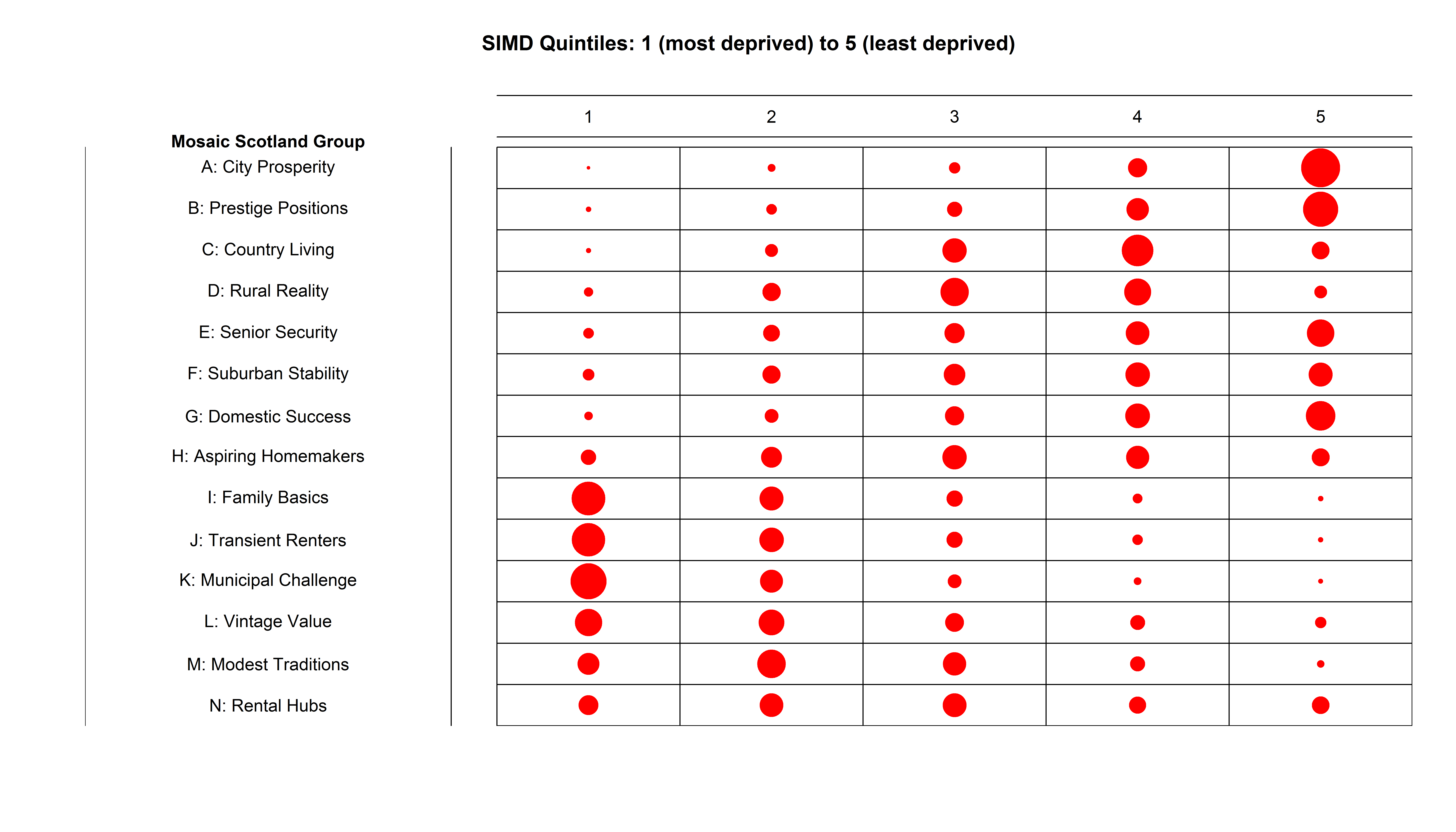


**Figure S1: Bubble chart for Experian Mosaic Scotland groups by SIMD Quintiles.** Dot size is proportion proportional to the percentage cell frequency.


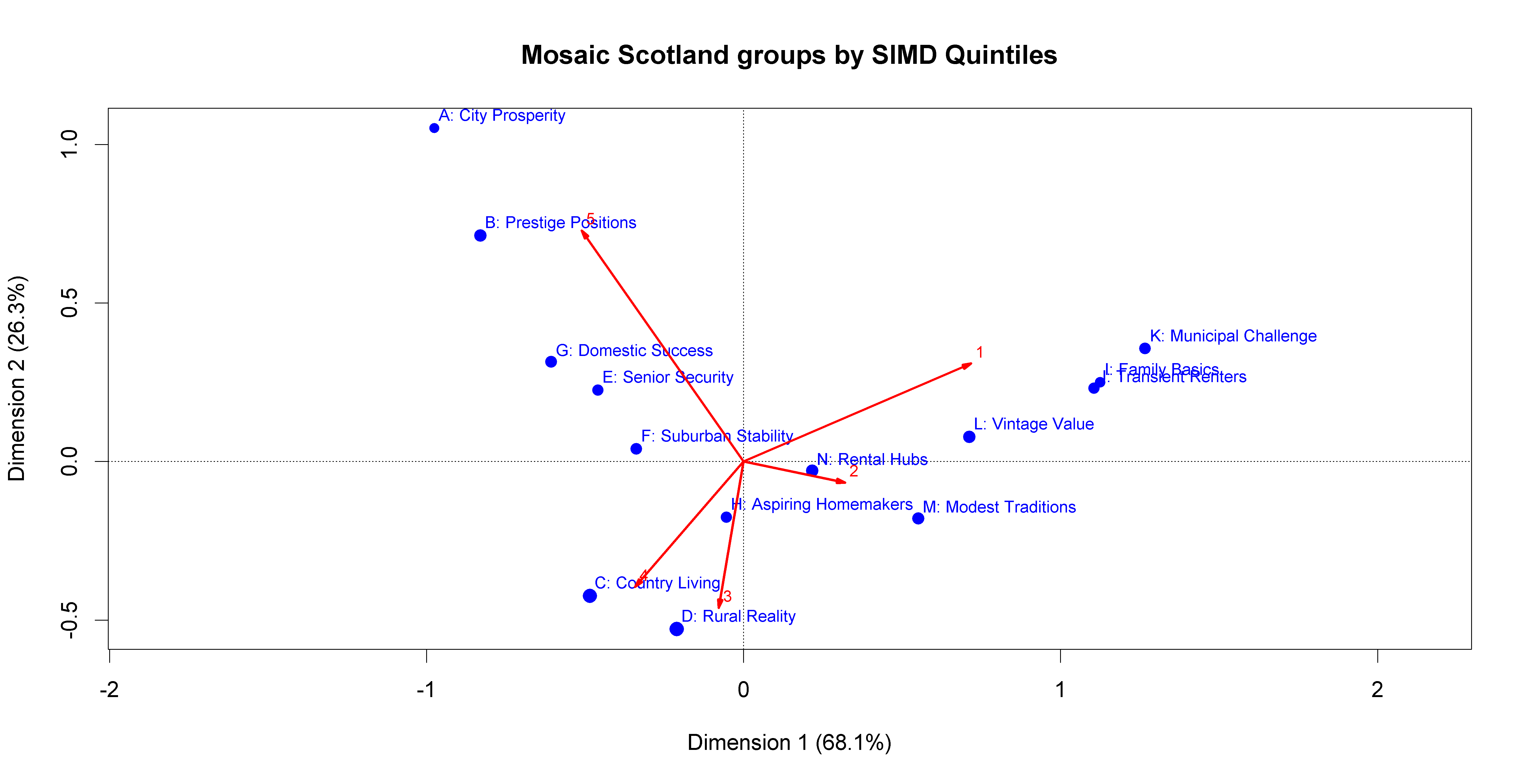


**Figure S2: Correspondence Analysis Biplot of Mosaic Scotland groups in correlation with levels of measures of deprivation.** The redlines represent the deprivation quintiles.
